# The population-based Barcelona-Asymptomatic Intracranial Atherosclerosis Study (ASIA): rationale and design

**DOI:** 10.1186/1471-2377-11-22

**Published:** 2011-02-17

**Authors:** Elena López-Cancio, Laura Dorado, Mónica Millán, Silvia Reverté, Anna Suñol, Anna Massuet, María Mataró, Amparo Galán, Maite Alzamora, Guillem Pera, Pere Torán, Antoni Dávalos, Juan F Arenillas

**Affiliations:** 1 Department of Neurosciences, Hospital Universitari Germans Trias I Pujol, Universitat Autònoma de Barcelona, Badalona, Barcelona, Spain; 2 Magnetic Resonance Unit, Hospital Universitari Germans Trias I Pujol, Universitat Autònoma de Barcelona, Badalona, Barcelona, Spain; 3 Department of Psychiatry and Clinical Psychobiology, Universitat de Barcelona, Barcelona (Spain; 4 Department of Biochemistry, Hospital Universitari Germans Trias I Pujol, Universitat Autònoma de Barcelona, Badalona, Barcelona, Spain; 5 Primary Healthcare Research Support Unit Metropolitana Nord, ICS-IDIAP Jordi Gol, Mataró, Barcelona, Spain; 6 Neurology Service, Stroke Unit, Hospital Clínico Universitario, Valladolid, Spain

## Abstract

**Background:**

Large-artery intracranial atherosclerosis may be the most frequent cause of ischemic stroke worldwide. Traditional approaches have attempted to target the disease when it is already symptomatic. However, early detection of intracranial atherosclerosis may allow therapeutic intervention while the disease is still asymptomatic. The prevalence and natural history of asymptomatic intracranial atherosclerosis in Caucasians remain unclear. The aims of the Barcelona-**AS**ymptomatic **I**ntracranial **A**therosclerosis (ASIA) study are (1) to determine the prevalence of ASIA in a moderate-high vascular risk population, (2) to study its prognostic impact on the risk of suffering future major ischemic events, and (3) to identify predictors of the development, progression and clinical expression of this condition.

**Methods/Design:**

Cross-over and cohort, population-based study. A randomly selected representative sample of 1,503 subjects with a mild-moderate-high vascular risk (as defined by a REGICOR score ≥ 5%) and with neither a history of cerebrovascular nor ischemic heart disease will be studied. At baseline, all individuals will undergo extracranial and transcranial Color-Coded Duplex (TCCD) ultrasound examinations to detect presence and severity of extra and intracranial atherosclerosis. Intracranial stenoses will be assessed by magnetic resonance angiography (MRA). Clinical and demographic variables will be recorded and blood samples will be drawn to investigate clinical, biological and genetic factors associated with the presence of ASIA. A long-term clinical and sonographic follow-up will be conducted thereafter to identify predictors of disease progression and of incident vascular events.

**Discussion:**

The Barcelona-ASIA is a population-based study aiming to evaluate the prevalence and clinical importance of asymptomatic intracranial large-artery atherosclerosis in Caucasians. The ASIA project may provide a unique scientific resource to better understand the dynamics of intracranial atherosclerosis from its early stages and to identify new potential therapeutic targets for this condition.

## Background

Atherosclerosis is a systemic disease with multifactorial etiology now considered the primary cause of morbidity and mortality in developing countries.

Large artery intracranial atherosclerosis disease (ICAD) is a major public health problem as it is probably the major cause of stroke worldwide and consequently, a main cause of long-term disability and mortality [1]. Accumulating evidence suggests that ICAD can also contribute to the development of cognitive impairment and Alzheimer disease although more studies are needed to establish this association [2-5]. Even so, it is a relative neglected frontier [1]. Despite the extended use of non invasive diagnostic techniques as transcranial Doppler (TCD), transcranial color-coded-duplex (TCCD), magnetic resonance angiography (MRA) or computed tomography angiography ICAD is an infradiagnosed and understudied disease when compared to extracranial atherosclerosis.

When it turns to symptomatic, intracranial atherosclerosis is a dynamic and aggressive disease. The rate of stroke recurrence is high (up to 18% in >70% stenosis) despite medical treatment [6-10], and the best therapy for symptomatic intracranial atherosclerosis still remains unknown [11]. In this context, there is a need to increase our knowledge about basic mechanisms and dynamics of intracranial atherosclerosis progression from its preclinical stage.

Intracranial atherosclerosis is not an isolated disease, but related to generalized atherosclerosis affecting other territories as carotid, coronary or peripheral artery disease [12,13]. One essential aim in primary prevention is to find tools to improve the evaluation of "the vulnerable patient" [14,15]. Classic vascular risk functions (as Framingham) are now being combined with new markers as carotid intima-media thickness, ankle-arm index or CRP values [16] to assess individual vascular risk. Therefore, the identification of new blood, genetic or instrumental biomarkers for asymptomatic atherosclerosis may be crucial to predict and prevent future ischemic events.

Traditional approaches have attempted to target ICAD when it is already symptomatic so there is a lack of studies in the asymptomatic stage. Population studies aimed to determine its prevalence and related vascular risk factors have only been developed in Asian populations, using transcranial Doppler to assess the presence of stenosis [17-20]. Therefore, the prevalence of asymptomatic intracranial atherosclerosis remains unknown in Caucasians.

In this context of uncertainty regarding the prevalence and clinical importance of asymptomatic intracranial atherosclerosis in Caucasians, we designed a prospective study called Barcelona-ASIA (ASymptomatic Intracranial Atherosclerosis), aimed (1) to determine the prevalence of asymptomatic intracranial atherosclerosis in a randomly selected Caucasian population with moderate-high vascular risk; (2) to study its prognostic impact on the risk of suffering future major ischemic events and/or cognitive decline; and (3) to identify predictors of the development, progression and clinical expression of this condition.

This article describes the protocol details of the Barcelona-ASIA study.

## Methods/Design

This is a population-based, prospective, long-term follow-up observational study that will include over a thousand randomly selected healthy subjects exposed to vascular risk factors and without history of stroke or ischemic heart disease. The study will have two phases, cross-sectional and longitudinal. In the first cross-sectional phase, all study subjects will undergo an extensive clinical, laboratory, ultrasound, neuropsychological and neuroimaging protocol in order to determine the prevalence of asymptomatic intracranial atherostenoses among the study population and to identify clinical, biological and genetic associated factors. In the second phase, a long-term clinical and TCCD follow-up will be performed with the purpose of determining the impact of asymptomatic intracranial atherosclerosis on the incidence of major vascular events and cognitive impairment.

### Subject selection

This study will be carried out in the Germans Trias i Pujol University Hospital, a public health tertiary centre of the Barcelonès Nord and Maresme region (Catalonia, Spain), and it is coordinated with the regional Primary Health Care network. The protocol has been approved by the Ethics Committee of our institution.

A rural and urban population of approximately 600.000 residents integrates our metropolitan area. This entire population is registered in a database of the Primary Care Information Technology System (SIAP). A sample of 3010 subjects older than 50 years was randomly selected in 2007 within the PERART study, an ongoing trial that aims to estimate the prevalence and prognosis impact of the peripheral artery disease in our population. Selection of the PERART cohort is described in detail elsewhere [21]. Framingham and REGICOR (Framingham calibrated for Spanish population [22]) functions were previously calculated in all these participants at the initial visit of PERART study.

The ASIA cohort derives from this PERART cohort. From the initial PERART sample of 3010 subjects, we will evaluate the 1503 subjects who met the following inclusion criteria: (1) No history of stroke or transient ischemic attack; (2) No history of coronary disease; (3) Exposure to a light-moderate-high vascular risk, assessed by a REGICOR≥5 (which is equivalent to a Framingham > 10); (4) Absence of institutionalization, severe disability or previous chronic neurological/psychiatric disease.

**Figure 1:**
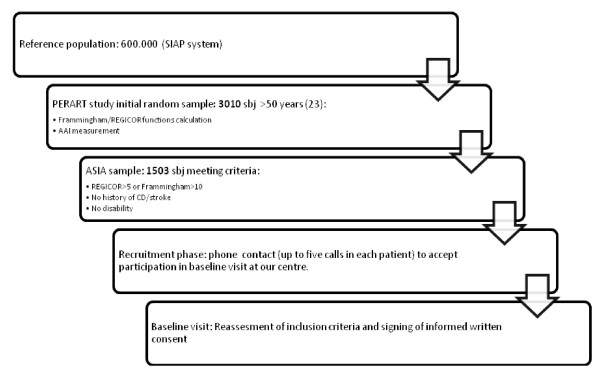
**ASIA study sample selection**. The diagram shows the sample selection in ASIA study from the reference population. SIAP system: Primary Care Information Technology System; sbj: subjects; AAI: ankle-arm index; CD: coronary disease.

First, on February 2007 a letter was sent to all 1503 selected participants warning them about the study and the chance of being called to take part in it. They were given a 15-day period to express their refusal to collaborate in the study, but no patient denied participation at that time. Subsequently, all possible participants will be contacted by phone (up to five calls) during the recruitment phase. A subset of this initial sample may reject to take part in the study when being informed about the conditions of the study during this phone call. Finally, among the subjects who will accept to come to our hospital for the baseline visit, some will be excluded after reassessment of inclusion criteria (Figure 1). We estimate that about 1000 subjects may complete the study, assuming a 10% of exclusions and a 20% of not-accepting participants.

### Baseline procedures at initial visit in our centre (Figure 2)

**Figure 2:**
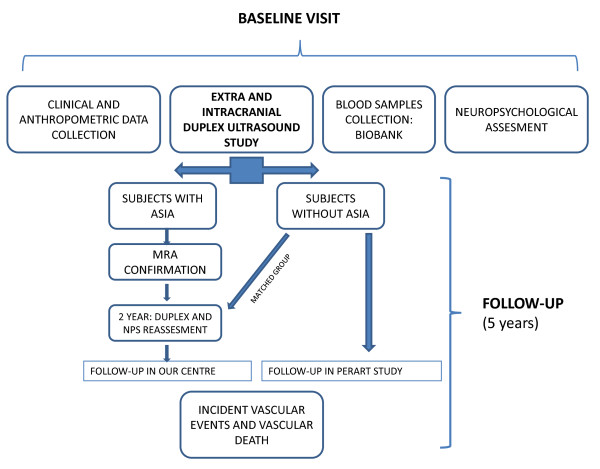
**ASIA study flow chart**. The diagram shows baseline procedures and follow-up in ASIA study. Subjects with asymptomatic intracranial atherostenoses (ASIA) in basal ultrasound study will undergo a Magnetic Resonance Angiograph (MRA) and will be followed annually in our centre. Two years after inclusion a new duplex and NPS (neuropsychological) study will be performed. Subjects without ASIA will be followed in PERART study.

1. Signing of informed written consent

**2. Clinical data collection**. Clinical data of every subject will be collected in a questionnaire specifically designed for this study:

◦ Sociodemographic variables: age, sex, socioeconomic status (monthly income in the family unit), education status (years of schooling, grade), employment (active, unemployed, retired, disqualified, housewife).

◦ Vascular risk factors: smoking habit (never, current, former), alcohol intake (g/day), physical activity level (sedentary, home-activity, outdoor activity and grade, walked Km/day in the last week), personal and family history of hypertension, diabetes mellitus and dyslipidemia.

◦ Current drug intake (platelet inhibitors, anticoagulants, lipid-lowering drugs, antihypertensives, hypoglycemic agents, antideppresants, etc).

◦ Vascular events presented between inclusion in the PERART study and baseline visit at our centre will be recorded: angina, myocardial infarction, intermittent claudication. Incidental stroke before the ASIA baseline visit was an exclusion criteria.

◦ Anthropometric variables: height, weight, waist circumference, current systolic/diastolic blood pressure values in both arms

◦ Ankle-arm index (AAI) was measured in all participants in PERART study as previously described (25).

◦ Others: Personal history of anxiety or depression, pharmacologic treatments in the moment of inclusion.

**3. Biological and genetic studies**. Twelve serum, 2 citrated plasma, 2 plasma-EDTA and 2 total blood cryotubes will be collected, processed and stored in each subject at baseline visit after a minimum of 12 fasting hours. After centrifugation at 3500 rpm and 4°C for 15 minutes, serum or plasma will be blind coded and stored at -80°C until analyzed.

**4. Ultrasound protocol**. All the Duplex studies will be performed in the same ultrasound lab (Neuroscience Department of Germans Trias i Pujol Hospital) using a General Electrid Vivid/Pro (GE Vingamed Ultrasound, Horten, Norway), by two experienced neurologists. First, a cervical study will be performed to detect presence and severity of atherosclerotic plaques in carotid arteries and origin of vertebral arteries, and to measure the intima-media thickness (IMT). Later, a transcranial duplex will be set to examine bilateral intracranial carotid artery (ICA), middle cerebral artery (MCA), anterior cerebral artery (ACA), posterior cerebral artery (PCA), vertebral artery (VA) and basilar artery (BA). All studies will be performed with subject in supine position.

**a. Extracraneal study: **It will be performed using a linear transducer 5-15 Hz (predefined parameters: 8.5 MHz, 4 cm focus, FPS 63.8).

◦ **Carotid assessment: **First, a high resolution B-mode study will be conducted in longitudinal and cross-sectional planes over the whole visible length of the following arterial segments: proximal and distal common carotid artery, carotid bifurcation and internal carotid artery. The most pathologic areas in every arterial segment will be analyzed and images stored.

▪ Atherosclerotic plaques. According to Manheim consensus criteria, atherosclerotic plaques are defined as follows: focal structure encroaching into the arterial lumen of at least 0.5 mm or 50% of the surrounding IMT value, or demonstrating a thickness > 1.5 mm as measured from the media-adventitia interface to the intima-lumen interface [23]. Atherosclerotic plaques will be further characterized by the following criteria:

• Plaque surface: regular smooth, irregular, ulcerated [24]

• Echogenicity: anechogenic, hypo-anechogenic, hypo-isoechogenic, iso-hyperechogenic.

▪ IMT. According to Manheim consensus criteria, IMT is defined as a double-line pattern (the interface between lumen-intima and interface between media-adventitia) visualized by B-mode sonography on both walls of the carotid arteries in a longitudinal image. IMT will be measured in regions without atherosclerotic plaque, preferably in CCA, in the far wall and more than 10 mm away from carotid bifurcation. Two values will be obtained: manual measurement of maximum IMT and automated measurement of mean IMT.

Next, Doppler spectrum analyses will be performed to assess the grade of stenosis depending on the systolic peak velocity [25]: <50%: <125 cm/s; 50-70%: 125-230 cm/s; >70%: > 230 cm/s. Significant carotid atherosclerosis will be considered when the stenosis is ≥ 50%. In stenosis < 50% the percentage of luminal obstruction in axial plane will be calculated.

◦ **Vertebral arteries**: Extracranial vertebral arteries will be studied in the following segments: V2 segment (longitudinal insonation plane, framed by acoustical shadow of transverse processes); V0/V1 segment (origin from subclavian artery); and V3 segment (transverse insonation below mastoid bone, comma-shaped). We will characterize vertebral arteries following spectrum analyses [26] as normal, stenosis >50% (SP >125 cm/s) or occlusion (absence of telediastolic flow).

**b. Transcranial color-coded-duplex (TCCD) study**: It will be performed using a 1.6-3.2 MHz transducer via transtemporal and transforaminal windows (patient in supine position) to evaluate circle of Willis and its branches, following consensus recommendations for an optimal exploration [27]. Each large cerebral artery will be investigated by spectral Doppler sonography with the color-coded Doppler signal used as a "road map". Flow direction, peak systolic (PSV), mean flow (MFV) and end-diastolic (PDV) velocities, pulsatility index (PI) and resistance index (RI) will be noted for every insonated artery. Angle correction will be performed when the Doppler sample volume is located within a straight vessel segment of at least 15-20 mm. By transtemporal bone window, we will study intracranial carotid artery (ICA) in axial and coronal planes, middle cerebral artery (M1 and M2 segments), anterior cerebral artery (A1 segment) and posterior cerebral artery (P1 and P2 segments). By transforaminal bone window we will study vertebral artery (V4 segment) and basilar artery (BA). If there is an insufficient acoustic window in transcranial examination, an enhance contrast agent will be used intravenously (Sonovue®)

### Intracranial stenosis definition and grading

**Figure 3:**
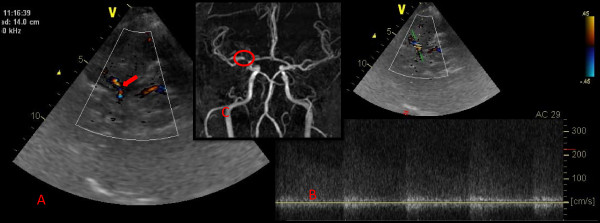
**Example of right middle cerebral artery (MCA) stenosis**. In left image (A) TCCD color mode shows an area of narrowing and color aliasing in the proximal segment of right MCA (arrow). In the right image (B), an spectral flow analyses is set to determine systolic peak velocity and appearance of low-frequency, high-intensity Doppler signals. In the middle superimposed image (C), a magnetic resonance angiogram (MRA) made in the same subject shows a flow gap in right MCA (circle) corresponding to a high-grade stenosis.

An intracranial stenosis will be diagnosed following previous published criteria. First, using color-coded mode as a road map we will assess flow direction and presence of segmental color aliasing phenomena in all arteries. Then, we will determine the presence of an intracranial stenosis if the spectral analysis shows a focal increase of PSV and/or PDV higher than the mean value + 2 SD for the corresponding cerebral artery or low-frequency, high-intensity Doppler signals, spectral widening or musical murmurs [28]. An example of intracranial stenosis is illustrated in figure 3. ICA stenosis will be diagnosed following published reference velocity values and recommendations, assessing not only differences in velocities between the two sides but also looking for activation of collaterals (anterior and/or posterior communicating arteries) and/or flow repercussion in extracranial internal carotid [29-31]. Following systolic peak criteria MCA stenosis will be graded into low-grade (140-209 cm/s), moderate (210-279 cm/s) and high-grade (>280 cm/s) [32]. For the rest of intracranial arteries we will follow cut-off values of PSV for < 50% and ≥ 50% stenosis [33]: ≥120/≥155 cm/s for anterior cerebral artery; ≥100/≥145 cm/s for posterior cerebral and basilar arteries and ≥90/≥120 cm/s for vertebral artery. Furthermore, when a focal increase of velocity is detected, the proximal and distal vessel segments will be evaluated (pre-stenotic and post-stenotic flow patterns in the upstream and downstream vessel segments) and potential collateral pathways will be considered in order to assess hemodynamic repercussion. Number, location and severity of intracranial stenosis will be recorded in every subject.

### Neuropsychological assessment

Neuropsychological studies will be performed at baseline visit in our centre after accepting and signing specific informed written consent. Neuropsychological studies will be performed by neuropsychologists blinded to neurosonology study results. General cognitive status will be measured using the Mini Mental State Examination. Depressive symptoms will be assessed with the Geriatric Depression Scale with scores higher than 5 being indicative of probable depression. Cognitive measures will assess executive functions, attention, verbal and visual memory, visuoconstructive abilities, speed/visuomotor coordination and language.

### Magnetic Resonance (MR) and MR-angiography (MRA) study

A complete MR will be conducted to patients with stenosis detected by TCCD if they have no contraindication. All explorations will be performed with the same 1.5T MR (Intera, Philips), with an echo-planar capacity of 25 m Teslas/m and time-rises of 300-350 microseconds. The MRA will be performed by a time-of-flight (TOF) sequence, using 1.5 mm section slides, 200-mm field of view, 200 × 512 matrix, and 7-11 minutes of acquisition. Maximal intensity projection (MIP) reconstructions will be set.

All MR studies will be performed in a 6 months maximum period from baseline inclusion visit (sonographic study). MRAs will be analyzed by a neuroradiologist blind to clinical and sonographic data. Intracranial stenosis will be defined as flow irregularity and focal narrowing >50% in luminal reduction affecting the main cerebral arteries.

### Primary prevention strategies

**- Vascular risk factors control (obesity, hypertension, dyslipidemia, hyperglycemia, sedentarism, smoking habit...)**. The family doctor will assume their control, after receiving a brief report of the neurosonolgical study and the clinical data obtained in the baseline visit in our centre.

**- Antiplatelet treatment: **After neurosonological study, antithrombotic treatment (300 mg of aspirin or 75 mg of clopidogrel-if there is intolerance/allergy to aspirin-) will be recommended according to AHA/ASA primary prevention guidelines [34] in the following cases: asymptomatic carotid stenosis > 50%; significant stenosis of extracranial vertebral arteries and/or asymptomatic intracranial stenosis.

**- Carotid revascularization: **Following the Asymptomatic Carotid Atherosclerosis Study (ACAS) [35], carotid revascularization (endarterectomy or endovascular) will be recommended in carotid stenosis > 60%, performed by surgeons or interventional neuroradiologists with periprocedural complication rates lower than 3%.

### ASIA clinical and image database

Clinical data will be recorded in CRFs. Blood samples will be processed, frozen and stored in our biobank. Images obtained in neurosonological studies will be stored in a specific workstation (Echo Pack) to be analyzed afterwards. A prospective electronic database will be created with clinical, neurosonological and laboratory variables of the cross-over and longitudinal phases of the study.

### Prospective follow-up (cohort study)

Participants will be followed-up annually for 5 years by primary care physicians of the PERART study to document the incidence of vascular events, therapeutic compliance and control of vascular risk factors. The primary endpoint will be the combined incidence of any major vascular event: acute myocardial infarction or angina requiring hospitalization, ischemic stroke, hemorrhagic stroke and vascular death.

Subjects with intracranial stenosis at baseline will be controlled in the Germans Trias i Pujol University Hospital annually. A new neurosonological and neuropsychological study will be performed two years after inclusion to study progression or regression of the intracranial lesions and cognitive decline. In addition, a matched group of subjects without baseline ASIA will be studied. Vascular events will be adjudicated by an external monitoring committee comprising of two neurologists and one cardiologist.

### Satatistical considerations

Statistics will be performed with the SPSS 18.0 statistical package. Quantitative variables will be compared with the Student's t test and analysis of variance will be performed, using the corresponding non parametric tests when necessary. Chi squared test will be used for comparisions of categorical variables. In the cross-over phase, multiple logistic regression models will be performed to identify variables independently associated with the presence of asymptomatic intracranial atherostenoses. In longitudinal phase, survival analysis for the combination of major vascular events and for each vascular event will be performed with the Kaplan-Meier curves according to the presence/absence of intracranial stenosis. Cox multivariate regression models will be used to compare the probability of having a vascular event in the follow up cohorts, adjusting for the necessary covariates. The relative risk (hazards ratios) will be given whit their corresponding 95% confidence intervals.

## Discussion

Despite of its relevant impact on public health and the development of new non-invasive diagnostic tests, intracranial atherosclerosis is still an understudied pathology. ICAD is the origin of 5-10% of strokes in Caucasians [36,37] and up to 50% in Asians [38,39]. Limited data about preclinical stage and natural history of ICAD are available in the general population. The only population studies to determine its prevalence in stroke-free individuals were developed in Asians without prospective follow-up to assess the risk of vascular events (21-25). These studies have some limitations as they evaluated intracranial stenosis only with transcranial Doppler, they did not use contrast agents and some of them only evaluated middle cerebral artery. There is too much variability in their methods to compare them and to establish a real prevalence. Our study is the first to evaluate the prevalence of asymptomatic intracranial atherosclerosis in Caucasians. As it is a cohort study we will also investigate the ASIA prognosis impact, not only in the appearance of cerebral events but also in coronary and peripheral artery disease, and in the development of vascular cognitive impairment.

We will determine clinical, instrumental (IMT and AAI measurements) and biological risk factors related to asymptomatic ICAD in order to identify factors associated with its progression and clinical expression. We will be able to analyze whether factors that have been already related to disease progression in its symptomatic stage, such as inflammatory markers, also pay a role in asymptomatic intracranial atherosclerosis [40-43]. Furthermore, we would evaluate the importance of using those risk factors as risk markers in terms of general vascular primary prevention.

The reasons that explain the racial and interindividual differences observed in the distribution of cerebral atherosclerosis affecting extra and intracranial arteries remain poorly understood. Differences in vascular risk factors profile, inflammatory markers, life-style and genetic susceptibility are proposed as possible answers but studies are contradictory. Our study could contribute to clarify this matter since we will evaluate extracranial and intracranial vasculature in each patient.

Finally, many studies are stressing the possible relationship between ICAD and cognitive impairment. A simultaneous protocol in collaboration with our neuropsychological team has been created to evaluate cognitive status in every subject of ASIA study. Consequently, the ASIA results may help to find out biological, sonographic and clinical factors related to the presence of vascular cognitive impairment in asymptomatic individuals.

Strengths of this study are: 1). Randomized, large sample population study that will provide a generalized estimation of prevalence of asymptomatic intracranial stenosis in Caucasians, which nowadays remains unknown; 2). TCCD assessment of all intracranial arteries (anterior and posterior circulation) in all subjects, use of contrast agents if insufficient acoustic window and MRA assessment in subjects with stenosis; 3). Complete cerebral neurosonology study in each patient (intracranial and extracranial) because an adequate interpretation of intracranial findings always requires a careful assessment of the influence of extracranial pathology on the intracranial hemodynamics; 4). A biobank will be created allowing investigation of molecular and genetic factors related to the presence and progression of intracranial atherosclerosis; 5). Long-term follow-up that will allow us to establish prognosis and related factors of incidental stroke and major vascular events, and to evaluate progression/regression of intracranial lesions.

We hope ASIA study will contribute to the better understanding of intracranial atherosclerotic disease dynamics and help us to identify new potential therapeutic and prevention targets for this condition.

## Competing interests

The authors declare that they have no competing interests.

## Authors' contributions

JFA conceived of and designed the study and is the principal investigator; AD is the Neurosciences Department of Germans Trias I Pujol Hospital director, participated in study design, have made a major revision of this manuscript and will provide all logistic support; ELC will carry out subject basal visits, ultrasonographic studies, database maintenance, subjects follow-up, coordination with other departments and wrote the initial draft of this article; LD will carry out patient basal visits and ultrasonographic studies; AS and SR will carry out the anthropometric and blood pressure measurements in basal visit and the processing and storage of biobank; AG will carry out molecular and bioquemistry studies. MM will coordinate all neuropsychological studies; MA, PT and GP provided randomized sample of subjects and clinical data from PERART study and will coordinate the follow-up study in primary care network. All authors read and approved the final manuscript.

## Funding support

This project is supported by the program of Promotion in the Biomedical Investigation and Health Sciences from the Carlos III Health Institute of the Spanish Health and Social Policy Ministry [PI070393]. Dr. López-Cancio is a neurologist granted with a Río Hortega research contract from the Carlos III Health Institute of the Spanish Health and Social Policy Ministry, co-financed by the Germans Trias i Pujol Research Institute Foundation. Neuropsychological studies are supported by the Juan de la Cierva research program of the Spanish Health and Social Policy Ministry [SEJ2006-15399/PSIC].

## Pre-publication history

The pre-publication history for this paper can be accessed here:

http://www.biomedcentral.com/1471-2377/11/22/prepub
